# Case Report: Autoimmune hepatitis in a patient with pseudohypoaldosteronism type 1—insights into a rare co-occurrence

**DOI:** 10.3389/fped.2025.1558179

**Published:** 2025-04-15

**Authors:** Hanan Al Thiabat, Jafar Alsheyyab, Israa Waleed Khalid, Rafeef Abdel Razzaq, Kamleh Barham, Hanaa M. Algharaibeh, Eyad Altamimi

**Affiliations:** ^1^ Department of Pediatrics and Neonatology, King Abdullah University Hospital, Faculty of Medicine, Jordan University of Science and Technology, Irbid, Jordan; ^2^ Faculty of Medicine, The Hashemite University, Zarqa, Jordan

**Keywords:** autoimmune hepatitis, pseudohypoaldosteronism type 1, salt loss, electrolyte disturbances, aldosterone

## Abstract

**Background:**

Pseudohypoaldosteronism (PHA) type 1 is a rare disease characterized by an end-organ unresponsiveness to mineralocorticoids, which results in salt loss from the kidney and impaired potassium and hydrogen secretion. It is subdivided into two main types: renal PHA and systemic PHA, which vary in presentation and severity.

**Case report:**

Our patient presented at the age of 1 month with fever and vomiting, electrolyte disturbances, hyponatremia, hyperkalemia, and metabolic acidosis. The infant was later diagnosed with PHA type 1 caused by a mutation of *SCNN1A*, and she had persistent elevation of liver enzymes, for which she was diagnosed with autoimmune hepatitis. She was initially treated with sodium supplements, sodium bicarbonate, and ion exchange resin (calcium polystyrene sulfonate); subsequently, prednisolone and azathioprine were added.

**Conclusion:**

We report a unique clinical presentation involving a patient who was diagnosed at the age of 1 month with PHA type 1 caused by a mutation of *SCNN1A* and who was diagnosed with autoimmune hepatitis. The coexistence of these two conditions could highlight a potential shared pathological pathway. Further research into the genetic and immunological links between these rare disorders is warranted.

## Introduction

Pseudohypoaldosteronism (PHA) type 1 is a rare disease that is characterized by an end-organ unresponsiveness to mineralocorticoids, which results in salt loss from the kidney and impaired potassium and hydrogen secretion (1). It can be primary (inherited) or secondary. The inherited form is subdivided into two main types: renal PHA and systemic PHA, which vary in presentation and severity.

Renal form PHA type 1 is caused by a mutation of mineralocorticoid receptor (MR) and is inherited as an autosomal dominant (AD) disorder. It manifests as metabolic acidosis, hyperkalemia, and salt loss during infancy (1). As opposed to the systemic forms, it often has a milder course and may eventually spontaneously remit over time (2).

The systemic form of PHA type 1 is caused by a mutation in the epithelial sodium channel (ENaC) and is inherited as an autosomal recessive trait (1). This impaired ENaC is not limited to the kidneys but is present in many organs, such as the salivary glands, sweat, and lungs (3). For this reason, infants may exhibit recurrent chest infections, skin changes, and a positive sweat test in addition to salt loss and hyperkalemia (3). 

The relationship between the systemic form of PHA type 1 and autoimmune hepatitis has not been previously addressed or mentioned, revealing a gap in our understanding that invites further exploration and investigation.

Herein, we describe a female patient who was diagnosed during her infancy with PHA type 1 caused by a mutation of *SCNN1A* who later presented with persistent elevated liver enzymes. Her liver biopsy confirmed the diagnosis of autoimmune hepatitis.

## Case description and diagnostic assessment

The patient was born at full term via cesarean section due to placental calcification, with a birth weight of 3,500 g. She was admitted on her second day of life to the neonatal intensive care unit (NICU) due to neonatal jaundice and was treated with phototherapy for 3 days. Upon discharge from the NICU, she was clinically and hemodynamically stable, receiving adequate nutrition through breastfeeding and formula supplementation. She was the second child of healthy, non-consanguineous parents, and her family history was unremarkable.

At 12 days old, she presented to the emergency department with non-projectile vomiting after each feed. The vomit consisted of milk without bile or blood. She appeared ill and dehydrated, with a depressed anterior fontanelle and delayed capillary refill. Her pulse rate was 185 bpm, and her axillary temperature was 37.8 °C. She had normal external female genitalia, and the rest of her physical examination was unremarkable.

Complete blood counts, kidney function test, C-reactive protein (CRP), and liver function test were ordered. The findings were remarkable for hyponatremia (Na: 126 mmol/L), hyperkalemia (K: 8 mmol/L), and metabolic acidosis (HCO_3_: 8 mmol/L). In addition, she had an elevated CRP (85 mg/dl), while her complete blood count, kidney function test, and random blood sugar were within normal limits. As part of a septic workup, cerebrospinal fluid, blood, and urine cultures were obtained, all of which returned negative.

She was admitted to the pediatric intensive care unit (PICU) and treated for metabolic acidosis, hyperkalemia, dehydration, and hyponatremia. Due to suspicion of congenital adrenal hyperplasia (CAH), hydrocortisone and fludrocortisone were initiated. Meanwhile, her hormonal profile was ordered and the results later showed the following: 17-hydroxyprogesterone 2.1 ng/ml, serum aldosterone >1,000 pg/ml, serum cortisol 204 nmol/L, adrenocorticotropic hormone (ACTH) 15.7 pg/ml, renin level 11.9 µIU/ml (normal range: 4.2–45.6), and karyotyping 46, XX (normal female karyotype).

At this stage, she did not respond to hydrocortisone or mineralocorticoid therapy, and her aldosterone levels remained markedly elevated. As a result, she was diagnosed with pseudohypoaldosteronism. She was discharged on Kayexalate (1 g/kg every 6 h), sodium bicarbonate (3.5 mEq/kg/day), and sodium chloride 3% (6 ml per feed, equivalent to 8 mEq/kg/day), with dosage adjustments based on her weight and electrolyte levels.

After discharge, she experienced recurrent episodes of vomiting, diarrhea, and respiratory symptoms, requiring multiple short-term hospital admissions.

Genetic analysis was subsequently performed, with genomic DNA extracted from an ethylenediaminetetraacetic acid (EDTA) blood sample using a standard protocol. Whole-exome sequencing, conducted using the xGen Exome Research Panel v2, identified a homozygous mutation in the *SCNN1A* gene (NM_001038.6:c.1360+2T>G). This variant, which had not been previously reported in other patients, results in a substitution at the splicing junction, causing an abnormal splicing effect. This mutation is predicted to lead to a loss of normal protein function through nonsense-mediated mRNA decay.

At 16 months of age, she presented with symptoms similar to her previous episodes, along with abdominal distension and hepatomegaly noted on physical examination, prompting further evaluation. Serial liver enzyme tests showed elevated levels, as detailed in Table 1, and an abdominal ultrasound revealed a homogeneous, mildly enlarged liver with a long axis of 10.7 cm without focal lesions.

**Table 1:** Patient's liver enzymes at baseline and at diagnosis of autoimmune hepatitis.

Liver function test	Baseline	At the age of 16 months	Normal range
Total protein (g/L)	71.77	66	60–80
Albumin (g/L)	47.2	33	38–54
Total bilirubin (μmol/L)	5	4	5–21
Direct bilirubin (μmol/L)	2.19	2	
Alkaline phosphatase (U/L)	235	283	142–335
Alanine transaminase (U/L)	20.9	132	0–33
Aspartate aminotransferase (U/L)	36.98	66	0–32
Gamma GT (U/L)	11.08	113	5–36

Viral serology was negative, but autoimmune markers were positive for anti-nuclear antibodies (ANA) and anti-smooth muscle antibodies (ASMA). A liver biopsy ultimately confirmed chronic hepatitis, characterized by a portal lymphoplasmacytic infiltrate, moderate interface hepatitis, and periportal fibrosis with periportal septa, classified as stage 2. These findings were consistent with autoimmune hepatitis type 1.

She was initially started on methylprednisolone (1 mg/kg once daily), azathioprine (2 mg/kg), and a proton pump inhibitor (PPI). The steroid dose was gradually tapered based on her clinical response and normalization of transaminase levels. She responded well to treatment and is now maintained on prednisolone (2.5 mg daily) and azathioprine, in addition to her other prescribed medications.

Currently, after almost 1 year of being diagnosed with autoimmune hepatitis, the patient is 2.5 years old, with a height of 83 cm (−1.8 SD) and a weight of 13.5 kg (0.35 SD). Her development is normal, and her kidney function and liver enzyme levels remain within normal limits.

## Discussion

In 1958, Cheek and Perry first described PHA type 1 as a defective renal tubular response to mineralocorticoids in infancy (4), caused by organ resistance to aldosterone, resulting in hyperkalemic metabolic acidosis with high serum aldosterone levels.

The inherited form is divided into two subtypes: the systemic form and the renal form. The renal form, which follows an autosomal dominant inheritance pattern, is primarily caused by inactivating mutations in the *NR3C2* gene, which encodes the aldosterone receptor (5). This results in milder signs and symptoms. In contrast, the systemic form is inherited in an autosomal recessive manner and is caused by mutations in the *SCNN1A*, *SCNN1B*, and *SCNN1G* genes, which encode the α, β, and γ subunits of ENaC, respectively. These mutations lead to impaired channel activity and a more severe phenotype (6).

The *SCNN1A* gene encodes the alpha subunit of ENaC (7). While ENaC is highly expressed in organs such as the lungs, kidneys, and pancreas, its expression in the liver is relatively low (7). According to data from the Human Protein Atlas, *SCNN1A* expression in the liver is minimal, with immunohistochemical staining showing a limited presence in both hepatocytes and cholangiocytes (8). Therefore, although *SCNN1A* is present in the liver, its low expression suggests that its role in hepatic function is likely to be minimal.

Our patient presented with hyponatremia, hyperkalemia, and metabolic acidosis. As a differential diagnosis for this electrolyte disturbance, possible causes include infection, congenital adrenal hyperplasia, or type 4 renal tubular acidosis.

The patient was initially managed with hydrocortisone, a mineralocorticoid, intravenous fluids, treatment for hyperkalemia, and antibiotics. Subsequent testing revealed elevated aldosterone, leading to a diagnosis of PHA. Further genetic testing revealed a homozygous mutation in the *SCNN1A* gene.

PHA is an extremely rare disorder, and the exact number of reported cases in the literature is not fully established. The estimated incidence of AR PHA is approximately 1:166,000, based on national statistics and birth rates in the Yorkshire and Humber region (1).

A 2017 systematic review examining 27 cases with follow-up data found that several patients needed gastrostomy tube placement to prevent salt-wasting crises. The majority of patients were of short stature, and while respiratory symptoms were common, they significantly decreased with age (9).

To the best of our knowledge, this is the first reported case of PHA type 1 associated with an autoimmune disease. This finding may enhance our understanding of the connection between these conditions and help guide the anticipatory management of PHA.

Autoimmune liver disease in children is also a rare condition characterized by chronic liver inflammation. It is classified into two types based on the presence of specific antibodies. Type 1 is associated with seropositivity for smooth muscle antibodies (SMA) and/or ANAs, while type 2 is characterized by the presence of liver-kidney microsomal type 1 (LKM-1) antibodies and/or anti-liver cytosol type 1 (LC-1) antibodies (10).

One proposed mechanism that could link these two diseases is suggested by a study examining the association between salt-losing tubulopathies (SLT) and altered immunity. In this study, 47 SLT patients with chronic salt depletion features (such as Bartter, Gitelman, or Epilepsy, Ataxia, Sensorineural deafness, and Tubulopathy Syndrome (EAST) syndromes) were enrolled. These patients also exhibited hyperaldosteronism and hypokalemic metabolic alkalosis, conditions commonly associated with renal magnesium wasting and hypomagnesemia. The study found that individuals with SLT had a higher prevalence of autoimmune diseases and an increased risk of bacterial infections (11).

In the same study, the authors investigated immunity in SLT patients. They analyzed CD4 subsets and IL-17 responses and found that SLT is associated with clinical features of immunodeficiency due to reduced IL-17 responses and that sodium depletion leads to changes in the ionic environment. This conclusion highlights the fundamental role of sodium and other extracellular ions in immune cell activation (11).

The main limitations of this case report are that it describes a single patient, and long-term follow-up data are not yet available.

## Data Availability

The original contributions presented in the study are included in the article/Supplementary Material, further inquiries can be directed to the corresponding author.
